# Efficient virus-induced gene silencing in apple, pear and Japanese pear using *Apple latent spherical virus *vectors

**DOI:** 10.1186/1746-4811-7-15

**Published:** 2011-06-10

**Authors:** Shintarou Sasaki, Noriko Yamagishi, Nobuyuki Yoshikawa

**Affiliations:** 1 Plant Pathology Laboratory, Faculty of Agriculture, Iwate University, Ueda 3-18-8, Morioka 020-8550, Japan

## Abstract

**Background:**

Virus-induced gene silencing (VIGS) is an effective technology for the analysis of gene functions in plants. Though there are many reports on virus vectors for VIGS in plants, no VIGS vectors available for *Rosaceae *fruit trees were reported so far. We present an effective VIGS system in apple, pear, and Japanese pear using *Apple latent spherical virus *(ALSV) vectors.

**Results:**

Inoculation of ALSV vectors carrying a partial sequence of endogenous genes from apple [ribulose-1, 5-bisphosphate carboxylase small subunit (*rbcS*), alpha subunit of chloroplast chaperonin (*CPN60a*), elongation factor 1 alpha (*EF-1a*), or actin] to the cotyledons of seeds by a particle bombardment induced highly uniform knock-down phenotypes of each gene on the true leaves of seedlings from 2~3 weeks after inoculation. These silencing phenotypes continued for several months. Northern blot and RT-PCR analyses of leaves infected with ALSV containing a fragment of *rbcS *gene showed that the levels of *rbcS*-mRNA drastically decreased in the infected apple and pear leaves, and, in reverse, *rbcS-*siRNAs were generated in the infected leaves. In addition, some of apple seedlings inoculated with ALSV vector carrying a partial sequence of a *TERMINAL FLOWER 1 *gene of apple (*MdTFL1*) showed precocious flowering which is expected as a knock-down phenotype of the silencing of *MdTFL1 *gene.

**Conclusions:**

The ALSV-based VIGS system developed have provides a valuable new addition to the tool box for functional genomics in apple, pear, and Japanese pear.

## Background

The infection of virus vector carrying sequences of plant genes triggers virus-induced gene silencing (VIGS) that results in the degradation of endogenous mRNA homologous to the plant genes through a homology-dependent RNA degradation mechanism [1,2]. Because VIGS offers an easy way to determine the functions of the genes in a short time, and it can also be applied to high throughput functional genomics in plants [1,3,4], the technology is an important tool for functional genomics in plants and used routinely for the analysis of gene function in many laboratories around the world. Though there are many reports on virus vectors for VIGS in plants, most are useful for the analysis of gene function in a limited range of dicot plants, e.g., *Arabidopsis thaliana, Nicotiana benthamiana*, *N. tabacum*, tomato, potato, legume species, cucurbits, and cassava etc, and monocot plants, barley and wheat [5-13], and their reliability and effectiveness depends on both plant species and virus vectors [4,14-17]. For these reasons, the development of reliable VIGS vectors for additional plant species will be very useful for the development of plant genomics [4,17-19].

Fruit tree crops have several problems for use of VIGS in functional genomics. First, there are few reports on effective VIGS-inducing virus vectors that can be used for fruit tree crops. *Citrus tristeza virus *and *Plum pox virus *vectors were reported for stable transient expression in citrus and apricot, respectively [20-22]. However, it is not evaluated whether the vectors are effective VIGS inducers and can be used for analysis of gene functions in fruit trees. *Grapevine virus A *(GVA) vector is the only virus vector reported for VIGS in a fruit tree, in which it was possible to silence the endogenous *phytoene desaturase *(*PDS*) gene in micropropagated grapevine plantlets [23]. On the other hand, no available virus vectors for VIGS were reported in *Rosaceae *fruit trees. Second, if the virus vectors were constructed from viruses which can infect fruit tree crops, it generally proved difficult to inoculate fruit trees with viruses [23,24] and, if possible, it takes a long time for systemic infection of inoculated virus and for analysis of the effects of virus infection in fruit trees, and the time generally exceeds the stability of virus vectors [20].

*Apple latent spherical virus *(ALSV), originally isolated from an apple tree, has isometric virus particles *c*. 25 nm in diameter, and it contains two ssRNA species (RNA 1 and RNA 2) and three capsid proteins (Vp25, Vp20 and Vp24) [25,26]. The virus did not induce any obvious symptoms in most of host species including apple. ALSV vectors have been constructed for the expression of foreign genes in plants [27] and used for analysis of virus movement and virus distribution in infected plant tissues [28,29]. Recently, we reported that ALSV vectors could be used for a reliable and effective VIGS among a broad range of plants, including legume and cucurbits species [5,30,31].

Here, we describe a rapid and easy VIGS system that effectively induces reliable VIGS of endogenous genes in the seedlings of apple, pear, and Japanese pear using ALSV vectors. To our knowledge, this is the first report on VIGS in apple, pear, and Japanese pear, and the method will be powerful tool for functional genomics in *Rosaceae *fruit trees.

## Results

### Virus-induced gene silencing (VIGS) of endogenous genes in the seedlings of apple, pear, and Japanese pear

**Figure 1:**
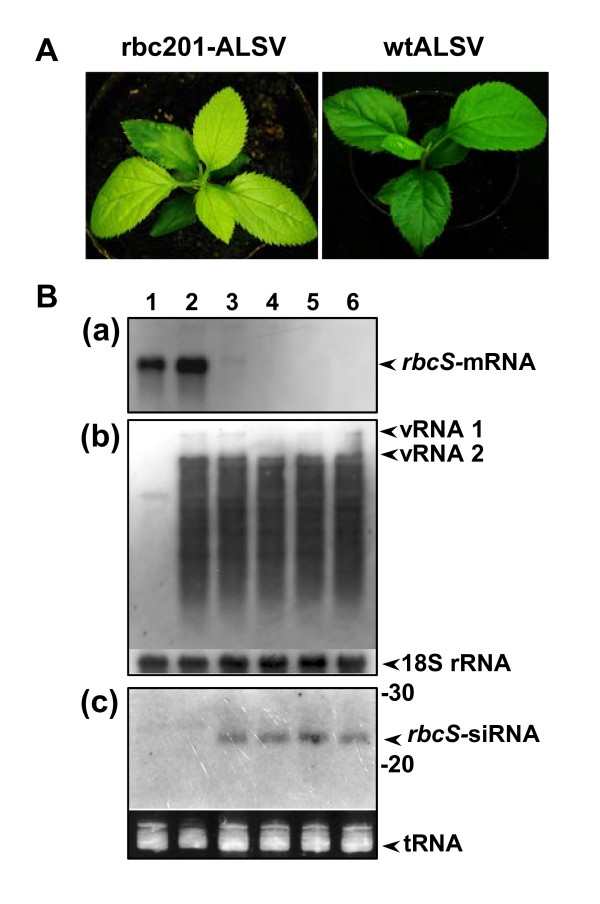
**VIGS of *rbcS *gene in apple by ALSV vector infection**. (**A**) Leaf chlorosis in an apple seedling infected with rbcS201-ALSV (left) and normal seedlings infected with wtALSV 45 days post inoculation (dpi). (**B**) Northern blot hybridization analysis of *rbcS-*mRNA (**a**), ALSV-RNAs (**b**) and *rbcS*-siRNAs (**c**) from leaves of the apple seedlings infected with rbcS201-ALSV. Lane 1, healthy control; lane 2, wtALSV-infected leaves; lanes 3-6, the apple seedlings infected with rbcS201-ALSV.

**Figure 2:**
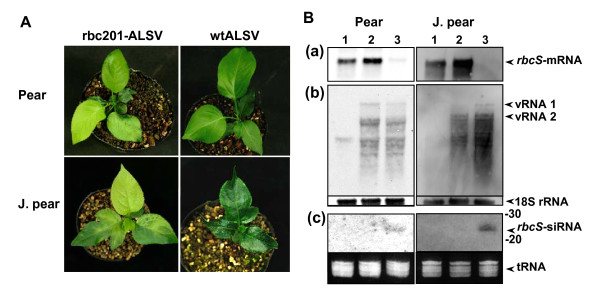
**VIGS of *rbcS *gene in pear and Japanese pear by ALSV vector infection**. (**A**) Leaf chlorosis in pear and Japanese pear (J. pear) seedlings infected with rbcS201-ALSV (left) and normal seedlings infected with wtALSV 45 dpi. (**B**) Northern blot hybridization analysis of *rbcS-*mRNA (**a**), ALSV-RNAs (**b**) and *rbcS*-siRNAs (**c**) from leaves of the pear and J. pear seedlings infected with rbcS201-ALSV. Lane 1, healthy control; lane 2, wtALSV-infected leaves; lanes 3, the seedlings infected with rbcS201-ALSV.

Because the sequences of widely used VIGS markers, phytoene desaturase (*PDS*) and a subunit of magnesium chelatase (*SU*), were not available in apple, we first targeted *rbcS *gene in apple, pear, and Japanese pear to investigate whether ALSV vectors could act as effective inducers for the silencing of endogenous genes in *Rosaceae *fruit trees. The *rbcS *gene fragment (201 bp) from apple was inserted into an ALSV-RNA2 vector, and the construct was inoculated into *C. quinoa *with pEALSR1, which is the cDNA infectious clone of ALSV-RNA1, by mechanical inoculation. The resulting virus was designated rbc201-ALSV. Total RNAs were extracted from rbc201-ALSV-infected *C. quinoa *leaves and then inoculated to the cotyledons of seeds just after germination by a particle bombardment. All inoculated seedlings (14 plants) of apple started to develop chlorosis on the 2^nd ^or 3^rd ^true leaves from 2 to 3 weeks post inoculation (wpi), and then newly developed true leaves showed a highly uniform chlorosis which is expected to be a knock-down phenotype of *rbcS *inhibition (Figure 1A and Table 1). RT-PCR indicated that all inoculated apple seedlings were infected with ALSV vectors (data not shown). Similar knock-down phenotypes was observed on all seedlings of pear (6 plants) and Japanese pear (6 plants) infected with rbc201-ALSV (Figure 2A and Table 1). The infected seedlings of apple, pear, and Japanese pear were severely stunted and the silencing phenotypes persisted for more than three months. The seedlings inoculated with a wild-type (wt) ALSV did not develop any viral symptoms nor a change of leaf color (Figures 1A and 2A).

**Table 1:** Phenotypic changes on seedlings of apple, pear, and Japanese pear infected with ALSV vectors

		Phenotypic changes		
		
Target gene	Plant species	Leaf	Growth	Flowering
-	Apple	Normal	Normal	-
*rbcS *	Apple	Chlorosis	Severe stunt	-
*rbcS *	Pear	Chlorosis	Severe stunt	-
*rbcS *	Japanese pear	Chlorosis	Severe stunt	-
*CPN60a *	Apple	Chlorosis	Severe stunt	-
*actin *	Apple	Distortion and curling	Slightly stunt	-
*EF-1a *	Apple	Deformity	Severe dwarf	-
*MdTFL1 *	Apple	-	-	Precocity

**Figure 3:**
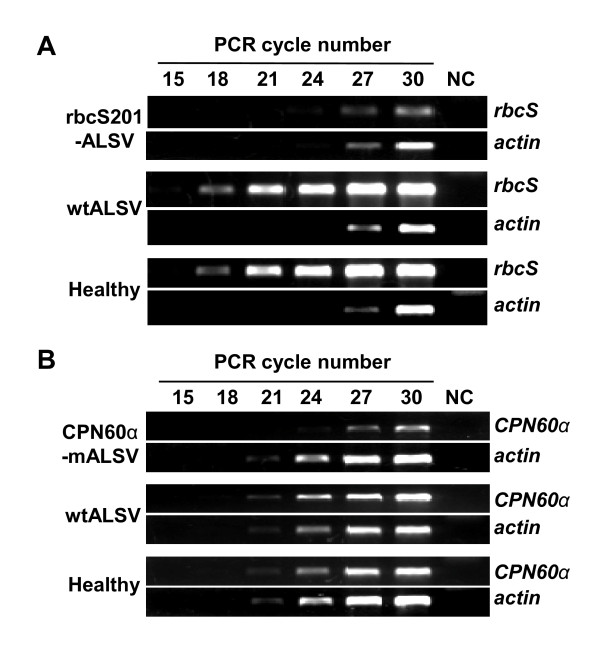
**RT-PCR analysis in apple seedlings infected with ALSV vectors**. RT-PCR analysis of *rbcS *(**A**) or *CPN60α *(**B**) mRNA levels in silenced (ALSV-vectors infected) and non-silenced (wtALSV-infected and healthy) leaves of apple seedlings. Lane NC represents the controls in which the reverse transcriptase-free RT reaction mix was used as a template in the reaction (30 cycles).

Total RNAs were extracted from leaves of apple, pear, and Japanese pear seedlings infected with rbc201-ALSV and analyzed by Northern blot hybridization and a semi-quantitative RT-PCR. Northern blot hybridization indicated that the levels of *rbcS*-mRNA drastically decreased in apple, pear, and Japanese pear leaves infected with rbc201-ALSV compared with those of non-infected and wtALSV-infected apple seedlings (Figures 1B and 2B). In reverse, *rbcS*-siRNAs were found from leaves of apple seedlings infected with rbc201-ALSV, but not from non-infected and wtALSV-infected plants (Figures 1B and 2B). In RT-PCR analysis, the levels of *rbcS-*mRNA were strikingly reduced in the leaves of seedlings infected with rbc201-ALSV compared with those in wtALSV-infected leaves and non-infected, healthy leaves (Figure 3A). These results demonstrated that the infection of rbc201-ALSV could induce VIGS of *rbcS*-mRNA in leaves of apple, pear, and Japanese pear seedlings.

**Figure 4:**
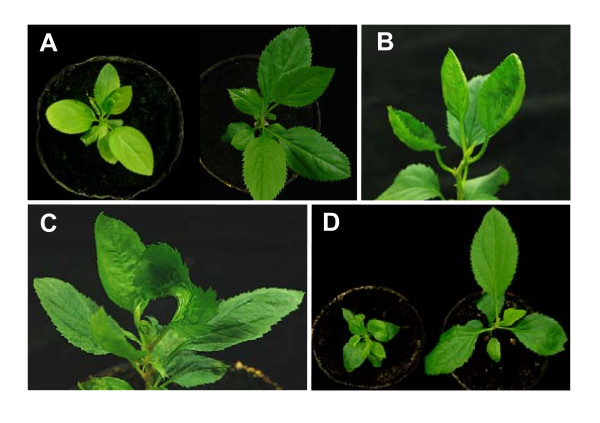
**VIGS of endogenous genes in apple seedlings by using ALSV vectors**. (**A**) Leaf chlorosis of the apple seedling infected with CPN60α-ALSV (left) and normal seedling infected with wtALSV (right) (68 dpi). (**B**) Distortion and curling of leaves of the apple seedling infected with actin-ALSV (50 dpi). (**C**) Deformity of leaves in the apple seedling infected with EF-1α-ALSV (61 dpi). (**D**) Severe dwarfing of the apple seedling infected with EF-1α-ALSV (left) and normal apple seedling infected with wtALSV (right) (46 dpi).

In the next experiments, *CPN60α, actin*, and *EF-1α *gene fragments (201bp) were inserted into ALSV-RNA2 vectors and the resulting viruses (CPN60α-ALSV, actin-ALSV, and EF-1α-ALSV) were inoculated to the cotyledons of germinated apple seeds as described above. Inoculated plants were assayed for virus infection 3 wpi by RT-PCR, indicating that all inoculated plants were infected with each virus vector. All apple seedlings (50 plants) inoculated with CPN60α-ALSV started to develop chlorosis on 2^nd ^or 3^rd ^true leaves from 2 to 3 wpi, and then newly developed true leaves showed a highly uniform chlorosis and the growth of silenced plants was then severely suppressed (Figure 4A and Table 1), similar to a knock-down phenotype of *rbcS *inhibition. A semi-quantitative PCR analysis indicated that the levels of *CPN60α-*mRNA was reduced in the leaves of apple seedlings infected with CPN60α-ALSV compared with those in wtALSV-infected and non-infected leaves (Figure 3B).

The actin-ALSV induced the distortion and curling of leaves of all infected apple seedlings (6 plants) about 3 wpi (Figure 4B and Table 1) and the silencing phenotype was maintained for more than 3 months. Similarly, all apple seedlings (6 plants) infected with EF-1α-ALSV developed the deformity of leaves and severe dwarfing (Figure 4C, D and Table 1).

### Precocious flowering in apple seedlings infected with ALSV carrying a fragment of MdTFL1

**Figure 5:**
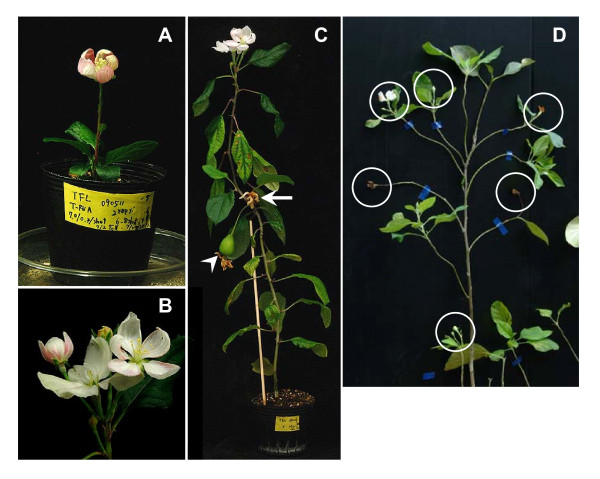
**VIGS of *MdTFL1 *gene in apple seedlings by ALSV vector infection**. (**A**) Precocious flowering of the apple seedling infected with MdTFL-ALSV (63 dpi). (**B**) Normal flowers in appearance with sepals, petals, stamens, and pistils on the seedling infected with MdTFL-ALSV. (**C**) The apple seedling infected with MdTFL-ALSV showing repeated flowering (124 dpi). An arrow indicated the first flower 68 dpi and an arrowhead showed a small fruit from the second flower after pollination with pollen from 'Golden Delicious' apple. (**D**) Flowering of the apple seedling infected with MdTFL-ALSV shown in (**c**) at the second year (605 dpi). Circled shoot apices changed to flowers.

Arabidopsis *TFL1 *is one of the genes that control flowering time in *A. thaliana *and plays a key role in the maintenance of the inflorescence meristem by preventing the expression of other genes in the shoot apical meristem [32,33]. We constructed the ALSV vector, MdTFL-ALSV, which carries a partial sequence of *MdTFL1*, a *TFL1*-like gene of apple [34], and inoculated the vector to apple seedlings. While most of plants infected with MdTFL-ALSV did not show any phenotypic changes and continued to grow vegetatively, ~10% of infected seedlings flowered from the 8 true leaf stage [1.5~2 months post inoculation (mpi)] to the 19 true leaf stage (Figure 5A, B, and 5C and Table 1). Flowers were normal in appearance with sepals, petals, stamens, and pistils (Figure 5B). When a flower was pollinated with pollen from 'Golden Delicious' apple, the pollinated flower set a small fruit containing fertile seeds (Figure 5C, an arrowhead), indicating the fertility of female gametes of a precocious flower. One of the flowered seedlings continues vegetative growth and flowering alternately in the current year (Figure 5C), and, after dormancy in winter season, the seedling restarted flowering on newly developed shoots in the second year (Figure 5D). Other flowered seedlings resumed vegetative shoot growth after one or two times of flowering. All apple seedlings infected with wtALSV and mock-inoculated seedlings grew vegetatively and did not produce flower buds under the same growth conditions, suggesting that the promotion of flowering was indeed due to MdTFL-ALSV infection.

**Figure 6:**
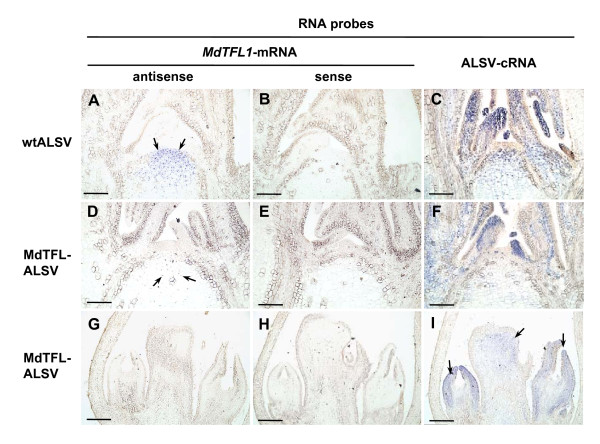
***In situ *hybridization analysis of apple seedlings infected with MdTFL-ALSV**. *In situ *hybridization analysis of *MdTFL1*-mRNA and ALSV-RNAs in the apical tissues of the apple seedlings infected with wtALSV (**A **to **C**) or MdTFL-ALSV (**D **to **I**). Three RNA probes, i.e., antisense RNA (**A**, **D**, and **G**) or sense RNA (**B**, **E**, and **H**) to *MdTFL1*-mRNA, and complementary RNA to ALSV-RNA (**C**, **F**, and **I**). Arrows in (**A**) and (**D**) indicate the hybridization signals showing the expression of *MdTFL1*-mRNA. Arrows in (**I**) indicate the presence of virus in floral organs of the apple seedling infected with MdTFL-ALSV.

We investigated the expression of *MdTFL1*-mRNA in the apical tissues of infected apple seedlings by *in situ *hybridization. The *MdTFL1*-mRNA has been detected in the cells of rib meristem of wtALSV-infected and healthy apple seedlings by an antisense probe, but not by a sense probe (Figure 6A, B). *In situ *hybridization analysis of apical tissues of three early-flowering seedlings infected with MdTFL-ALSV showed two patterns of expression of *MdTFL1*-mRNA. One is found in the infected seedlings No. 1 and No. 2, which continued vegetative growth after flowering once or twice. In these plants, the signals of *MdTFL1*-mRNA in the meristematic tissues was reduced compared with that in uninfected control and the signals were only found in small number of cells in the meristem (Figure 6D). The other is found in the infected seedling No.3 that continued vegetative growth and flowering alternately as described above. In three samples from the seedling No. 3, all shoot apical tissues already differentiated to flower buds and *MdTFL1*-mRNA was not detected at all in the apical tissues (Figure 6G, H). On the other hand, viral RNAs were distributed in the meristematic tissues in the shoot apical meristem as well as in all leaf primordia of wtALSV-infected seedling (Figure 6C) and the MdTFL-ALSV-infected seedlings No. 1 and 2 (Figure 6F), and floral organs of the MdTFL-ALSV-infected seedling No.3 (Figure 6I).

**Figure 7:**
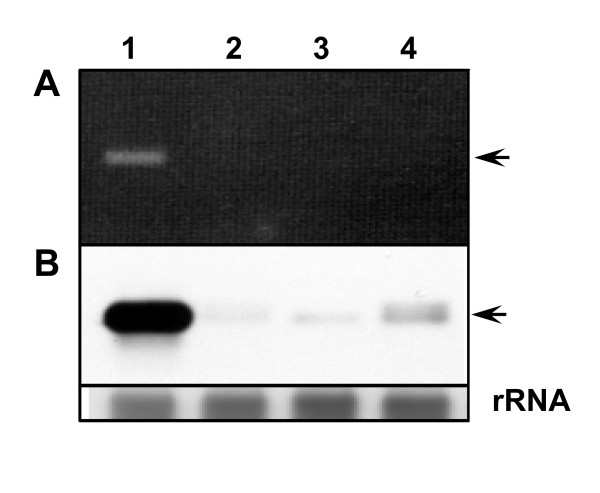
**Detection of *MdTFL1*-mRNA in apple seedlings infected with MdTFL-ALSV**. (**A**) RT-PCR analysis (ethidium bromide staining) and (**B**) RT-PCR- Southern blot hybridization for *MdTFL1*-mRNA in the apical tissues of the MdTFL-ALSV-infected apple seedlings. Lane 1, healthy; lane2-4, three shoot apices infected MdTFL1-ALSV. Arrows indicate the position of PCR product of *MdTFL1-*mRMA.

In addition, we analyzed the expression of *MdTFL1*-mRNA in the apical tissues of three MdTFL-ALSV-infected apple seedlings by RT-PCR-Southern blot hybridization. These seedlings continued to grow vegetatively without flowering although the plants were systemically infected with MdTFL-ALSV. Figure 7 shows that *MdTFL1*-mRNA was drastically reduced in the apical tissues of plants infected with MdTFL-ALSV compared with non-infected apple seedling, suggesting that VIGS of *MdTFL1*-mRNA was induced in the shoot apex of infected apple seedlings showing vegetative growth without flowering.

### Efficiency of VIGS in apple seedlings by ALSV vectors with inserts of different length

To investigate the influence of insert length on the efficiency of VIGS in apple, ALSV vectors carrying different lengths of *rbcS *gene (201 bp; rbcS201-ALSV, 102 bp; rbcS102-ALSV, and 51 bp; rbcS51-ALSV) were inoculated to the cotyledons of germinated seeds of apple. The seedlings infected with rbcS102-ALSV or rbcS51-ALSV all showed a highly uniform chlorosis similar to those infected with rbcS201-ALSV as described above (data not shown). Northern hybridization indicates that the amounts of *rbcS*-mRNA decreased in apple leaves infected with rbcS102-ALSV and rbcS51-ALSV, similar to those of rbcS201-ALSV-infected apple seedlings (data not shown). With *rbcS *gene, the 51, 102, and 201 bp sequences could suppress *rbcS *mRNA at the same level.

### Stability of the insert genes in ALSV vectors in apple seedlings

Evaluation of the effects of VIGS takes longer in fruit trees than in annual plants. To investigate the stability of the endogenous genes inserted to ALSV vectors in apple seedlings, total RNAs were extracted from the uppermost leaf of each vector-infected seedling at different periods after inoculation, and subjected to RT-PCR analysis to confirm whether vectors retained the insert sequences. As shown in Table 2 ALSV vectors maintained the inserts in 95%, 85% and 63% infected plants at 1.5 mpi, 3 mpi, and 6 mpi, respectively. Three seedlings infected with MdTFL-ALSV were further checked at prolonged periods. The virus in one seedling had lost *MdTFL *insert at 10 mpi, and viruses in other two plants maintained the sequences more than 12 mpi. Unfortunately, one seedling died at 12 mpi, but another apple seedling retains *MdTFL *insert (more than 24 months) and continues flowering until now.

**Table 2:** Stability of the endogenous genes inserted into ALSV vectors in apple seedlings

		No. of plants infected with ALSV maintained inserts/tested		
		
ALSV vectors	Sizes of Inserts (bp)	1.5 mpi	3 mpi	6 mpi
rbcS201-ALSV	201	4/4	4/4	0/2^a^
rbcS102-ALSV	102	5/5	5/5	-^a^
rbcS51-ALSV	51	5/5	5/5	5/5
CPN60a-ALSV	201	11/12	7/12	7/12
actin-ALSV	201	5/5	5/5	3/5
EF-1a-ALSV	201	4/5	4/5	2/5
MdTFL-ALSV	201	3/3	3/3	3/3
Total (%)		37/39 (95)	33/39 (85)	20/32 (63)

^a ^Two plants with rbcS201-ALSV and 5 plants with rbcS102-ALSV died before 6 mpi.

## Discussion

To date, there have been no effective silencing-inducing methods in *Rosaceae *fruit trees except the use of transgenic procedures. Successful transformation, however, is confined to a limited species and cultivars in *Rosaceae *fruit trees. It is also time-consuming and the transformation efficiency is very low [34]. In this study, we developed a rapid and easy VIGS system in the seedlings of apple, pear, and Japanese pear using the ALSV vectors. The ALSV vectors carrying apple *rbcS *gene fragment induced a highly uniform chlorosis (Figures 1, 2 and Table 1) which is expected to be a knock-down phenotype of *rbcS *inhibition [16]. The *CPN60α *gene was reported to be required for plastid division in *A. thaliana *and a null mutant in *CPN60α *resulted in an albino phenotype while a weaker mutation reduced chlorophyll levels [35]. The CPN60α-ALSV induced a highly uniform chlorosis on leaves of apple seedlings (Figure 4A and Table 1), suggesting that the *CPN60α *in apple has the same functions as those in *A. thaliana*. It is also reasonable to think that the deformity of leaves and severe dwarfing of the apple seedlings infected with EF-1α-ALSV (Figure 4C, D and Table 1) are due to the inhibition of the functions of *EF-1α *by VIGS.

As described in Introduction, there have been several problems for use of VIGS to analyze the functions of interesting genes in fruit trees. One is that fruit trees are generally insensitive or resistant to conventional mechanical inoculation [23,24,36]. Our finding that the cotyledons of apple seeds just after germination are very sensitive for virus inoculation enables efficient infection of ALSV vectors to apple seedlings [36]. In fact, by this method, almost all plants could be infected, and virus could be detected from the first true leaf just above the inoculated cotyledons. We think that the inoculation method may be applicable to the seeds of other *Rosaceae *fruit trees as well as any cultivars of apple, pear, and Japanese pear.

One of the characteristics of VIGS by ALSV vector in herbaceous plants is the induction of uniform silencing phenotypes and their persistency for several months in plants [5]. This was reproduced in the seedlings of apple, pear, and Japanese pear. For example, the apple seedlings inoculated with rbcS-ALSV started to develop chlorosis on the 2^nd ^or 3^rd ^true leaves from 2 to 3 weeks post inoculation, then newly developed true leaves showed a highly uniform chlorosis (Figure 1), and systemic silencing phenotypes persisted for more than three months in most infected plants. Thus, the VIGS system using ALSV vectors can induce VIGS in apple seedlings as efficiently and persistently as in herbaceous plants.

Apple has a long-juvenile period, generally lasts five to 12 years, during which flowering does not occur. It is difficult to reduce the juvenile phase of apple seedlings to < 2 years by agrotechnical approaches, though the long-juvenile period has been a serious contaminant for efficient apple breeding [37-39]. Kotoda et al. [34] reported that the co-suppression of *MdTFL1 *in apple, a *TERMINAL FLOWER 1 *homologous gene which acts as a repressor of flowering in *Arabidopsis thaliana*, induces the precocious flowering of the transgenic apples only 8 months after transfer to the greenhouse. In this study, we also showed that some of apple seedlings inoculated with ALSV vector carrying a partial sequence of *MdTFL1 *showed precocious flowering at the 8 true leaf stage (2 mpi). In *A. thaliana*, *TFL1 *is expressed in the center of the main lateral shoot inflorescence meristems and plays a key role in the maintenance of the inflorescence meristem [40,41]. *In situ *hybridization analyses of wtALSV-infected and healthy apple seedlings showed that *MdTFL1*-mRNA was expressed in the cells of rib meristem (Figure 6A), similar to that in *A. thaliana*. In contrast, the expression of *MdTFL1*-mRNA was reduced in shoot meristems of the early-flowering seedlings infected with MdTFL-ALSV (Figure 6D). This may be due to VIGS induced by MdTFL-ALSV infection, because ALSV can enter into meristematic tissue (Figure 6C, F) and silence the meristem-expressed genes as previously reported [5].

At present, it remains unknown why some plants infected with MdTFL-ALSV showed precocious flowering, whereas other did not. One possible reason is that the silencing of *MdTFL1 *gene is incomplete so that flowering was not induced in all infected plants. However, analysis by RT-PCR-Southern blot hybridization showed that *MdTFL1*-mRNA was drastically reduced in the apical tissues of MdTFL-ALSV-infected plants which continued to grow vegetatively without flowering (Figure 7).

Alternatively, genetic factors controlling floral initiation in apple seem to be more complex than those of herbaceous model plants [39,42-44]. We recently reported that the Arabidopsis *FLOWERING LOCUS T *(*FT*) expressed by the ALSV vector could induce early flowering in about 30% of infected apple seedlings [45]. However, expression of a *FT *homolog from apple (*MdFT1*) by the ALSV vector (MdFT-ALSV) did not induce early flowering in apple seedlings. When ALSV genome replicated in the cells of shoot apical meristem, MdFT1 protein must be translated from RNA2 of MdFT-ALSV in the cells of shoot apical meristem. As one possibility, a balance and timing of both *MdFT1 *and *MdTFL1 *expression may be important for the transition from the vegetative to the reproductive phase in apple.

As shown in Table 2 ALSV vectors had lost their inserts in 5%, 15%, and 37% in infected plants at 1.5 mpi, 3 mpi, and 6 mpi, respectively. Although the number of plants used for evaluation of vector stability containing different sizes of inserts is not enough, stability of inserts seems to depend on the sequences and the sizes of the gene fragments. It is worth noting that one of apple seedlings infected with MdTFL-ALSV maintains the insert sequence for more than 24 months and continues to show early-flowering phenotype (Figure 5D). This may open a possibility for functional analysis of genes in the reproductive phase of apple. We recently showed that ALSV could infect all parts of the ovule and is also present inside infected pollen grains [46].

A collection of expressed sequence tags (EST) and genome sequences of the domesticated apple have been reported [47,48]. Our rapid and effective VIGS-inducing system reported here might be a powerful tool for functional analysis of interesting genes in apple, e.g., resistant-related genes and genes involved in flower and fruit development etc, by combination with molecular data.

## Conclusions

In conclusion, we demonstrated that ALSV vectors induced highly uniform knock-down phenotypes of endogenous genes in *Rosaceae *fruit trees. When the cotyledons of seeds were inoculated with ALSV vectors by a particle bombardment, the silencing phenotypes appeared on the true leaves from 2~3 weeks after inoculation and continued for several months. Thus, the VIGS system developed here provide an easy way for functional genomics in apple, pear, and Japanese pear.

## Materials and Methods

### Construction of ALSV vectors

The sequences of the ribulose-1, 5-bisphosphate carboxylase small subunit (*rbcS*), the alpha subunit of chloroplast chaperonin (*CPN60α*), *actin*, and elongation factor 1 alpha (*EF-1α *genes were amplified from total RNA from apple leaves using primer pairs in Table 3. The amplified DNAs were cloned into pGEM^R^-T Easy vectors (Promega, Madison, USA), and all cloned sequences were confirmed by automated dye-terminator sequencing using an ABI 310 sequencer. To construct ALSV vectors, a DNA fragment was amplified by using a cloned DNA of each gene as a template using a primer pair containing *Xho *I and *Bam *HI sites in Table 1. The DNA product was double-digested with *Xho *I and *Bam *HI and ligated to pEALSR2L5R5GFP restricted with the same enzymes [30].

**Table 3:** Oligonucleotides used in this study

Primers	Sequences (5' - 3')	**Genebank accession no**.
Primers used for amplification of genes from apple		
rbcS(+)	^1^GACGAGAAAGCAGAGAGAGA^20^	L24497
rbcS(-)	^500^CGATGATACGGATGAAGGAT^481^	L24497
CPN60α(+)	^1248^ACTAATGACTCWGCTGGYGA^1267^	DL175273
CPN60α(-)	^2637^GTWGCRTTCTTKGCATCCTC^2618^	DL175273
actin(+)	^1^TCCTTCGTCTTGACCTTGCT^20^	DQ822466
actin(-)	^245^ACGGAATCTCTCAGCTCCAA^226^	DQ822466
EF-1α(+)	^45^ATGACCCTGCCAAGGAGGCT^64^	U80268
EF-1α(-)	^644^GAATCGACACAACATAAACT^625^	U80268
EXP2(+)	^52^ATGGCTTGAGCTGCGGGTCT^71^	AY083167
EXP2(-)	^651^ACAATTCACCCTTCACCATT^632^	AY083167
PIP1b(+)	^84^ATGGAAGGCAAGGAAGAGGA^103^	AB100870
PIP1b(-)	^673^AAAACGGTGTAGACGAGGAC^654^	AB100870
Primers used for ALSV vector construction		
rbcSXho(+)	TACATCTCGAG ^50 ^GGTACCGTGGCTACAGTT^67^	
rbcS201Bam(-)	TACATGGATCC ^250 ^AGGAAGGTAAGAGAGGGT^233^	
rbcS102Bam(-)	TACATGGATCC ^151 ^ATTGCTTTTTCTGGTGAC^134^	
rbcS51Bam(-)	TACATGGATCC ^100 ^TGGAGCAACCATTCTGGC^83^	
CPN60αXho(+)	TACATCTCGAG ^1864 ^ACTGACCAGAAGATTTCA^1871^	
CPN60αBam(-)	TACATGGATCC ^2064 ^GAGGAGAGCCTTTCTCCG^2047^	
actinXho(+)	TACATCTCGAG ^15 ^CTTGCTGGTCGTGACCTC^32^	
actinBam(-)	TACATGGATCC ^215 ^TTGGCCATCGGGAAGCTC^198^	
EF-1αXho(+)	TACATCTCGAG ^179 ^CTTACCAAGGTTGACAGG^196^	
EF-1αBam(-)	TACATGGATCC ^379 ^CTCAACGCTCTTGATAAC^362^	
EXP2Xho(+)	TACATCTCGAG ^252 ^AGAGCTGGAATTGTTCCT^269^	
EXP2Bam(-)	TACATGGATCC ^452 ^CCAGTTCTGGCCCCAGTT^435^	
PIP1bXho(+)	TACATCTCGAG ^405 ^ACCGCCGGCATCTCCGGT^422^	
PIP1bBam(-)	TACATGGATCC ^605 ^ATGGGCAACAGAGTTGGC^588^	
MdTFLXho(+)	TACATCTCGAG ^40 ^ATGAAAAGAGCCTCGGAG^57^	
MdTFLBam(-)	TACATGGATCC ^204 ^CACCAAAGTAAAGAAAGA^223^	

Numbers on sequences refer to positions in genes.

Restriction sites of *Xho *I and *Bam*HI were underlined

To construct ALSV vectors containing different sizes of *rbcS *gene, DNA fragments were amplified from a *rbcS*-cDNA clone using primer pairs, rbcSXho(+) and rbcS201Bam(-), rbcS102Bam(-), or rbcS51Bam(-) (Table 1). The DNA products were inserted to a RNA2 vector as described above, and the resulting vectors were designated pEALSR2L5R5 rbcS201, pEALSR2L5R5 rbcS1012, and pEALSR2L5R5 rbcS51, respectively.

The ALSV vector containing apple *TERMINAL FLOWERING 1-*like gene (*MdTFL*1) (Genbank/EMBL/DDBJ accession no. AB052994) was constructed as follows: MdTFL1 fragment was amplified using MdTFL1 full-length cDNA clone (pBSMdTFLfull#12, kindly supplied by Dr. N. Kotoda) as a template and primer pairs MdTFLXho(+) and MdTFLBam (-) (Table 1). The DNA product was ligated to an ALSV-RNA2 vector as described above.

The constructed vectors were purified from large-scale cultures of *Escherichia coli *JM109 using a QIAGEN plasmid Maxi kit (QIAGEN, Duesseldorf, Germany) and then mechanically inoculated to *Chenopodium quinoa *plants with pEALSR1 [27]. Ten to sixty percents (depending on vectors) of inoculated *C. quinoa *plants showed chlorotic symptom on upper leaves after two to three weeks. Leaves with symptoms were homogenized in 3 volumes of extraction buffer (0.1 M Tris-HCl, pH7.8, 0.1 M NaCl, 5 mM MgCl_2_) and reinoculated to *C. quinoa *plants. The infected leaves were used for RNA extraction.

### Plant materials and viral inoculation

Seeds from apple, pear, and Japanese pear were stored at 4 °C and germinated seeds were used for biolistic inoculation as described below.

Total RNAs were extracted from infected *C. quinoa *leaves by Tri reagent and inoculated to the cotyledons of germinated seeds by particle bombardment using a PDS-1000/He Particle Delivery System (Bio-Rad, Hercules, CA, USA) or a Helios Gene Gun system (Bio-Rad) as described by Yamagishi *et al*. [36]. After inoculation and acclimation, the seeds were sown in soil and grown in a growth chamber (25 °C, 16 h: 8 h light:dark photoperiod).

### RNA extraction, semi-quantitative RT-PCR, Northern blot hybridization, and RT-PCR-Southern blot hybridization

Total RNAs were extracted from infected apple leaves according to Gasic *et al*. [49] with slight modifications. Briefly, ca. 50 mg of apple, pear, or Japanese pear leaves was homogenized with 500 μl extraction buffer (2% [w/v] cetyltrimethylammonium [CTAB], 2% polyvinylpolypyrrolidone [PVP], 100 mM Tris-HCl [pH 8.0], 25 mM EDTA [pH 8.0], 2 M NaCl, 2% β-mercaptoethanol) in a Micro Smash MS-100 bead beater (TOMY, Tokyo, Japan). The homogenates were incubated at 65 °C for 15 min and then mixed with 500 μl chloroform for 2 min. After centrifugation at 10,000 rpm for 10 min, the aqueous phases were added to one-third volumes of 7.5 M LiCl and incubated at -80 °C for 30 min or -4°C overnight. After centrifugation at 14,000 rpm for 30 min, RNA pellets were washed with 70% ethanol. After DNase I treatment, phenol/chroloform extraction, and ethanol precipitation, RNA pellets were dissolved in RNase-free water at a concentration of 1 μg/μl. Small RNAs were extracted from apple leaves by using mir Vana™miRNA Isolation Kit (Ambion, TX, USA) according to Instruction Manual.

For RT-PCR, first strand cDNA was synthesized using 2 μg of RNA, oligo(dT) primer, and Rever Tra Ace reverse transcriptase (TOYOBO, Osaka, Japan). Semi-quantitative RT-PCR was conducted as described by Burton *et al*. [50]. PCR amplifications were performed for 15, 18, 21, 27, and 30 cycles using a primer pair, rbcS251(+) (5'-^251 ^CCCCTTTCTACCGAGTCCTT^270^-3') and rbcS(-) (Table 1) for rbcS gene and a primer pair, CPN(+) (5'-^2366^TCAGTTGAGCAGCTTGGT^2383^-3') and CPN(-) (5'-^2566^TATAACAGCAACTCCACC^2549^-3') for the *CPN60α *gene. Apple actin gene [a primer pair, actin (+) and actin (-) in Table 1] was used as an internal control.

To test the vector stability containing different size of insert, first strand cDNA was synthesized using 1 μg RNA, oligo(dT) primer, and Rever Tra Ace reverse transcriptase. PCR amplification was performed using 1 μl template cDNAs, a primer pair R2ALS+ primer (5'-^1362^GCGAGGCACTCCTTA^1376^-3') and R2ALS- (5'-^1524^GCAAGGTGGTCGTGA^1510^-3'), which were designed for the amplification of a specific region containing the insert sequence on the ALSV RNA2 genome. The PCR amplification was conducted as described by Yamagishi *et al*. [45].

For Northern blot analysis of ALSV-RNAs and *rbcS*-mRNA, total RNAs were separated on a 1.5% agarose gel containing 6% formaldehyde and transferred to a Hybond-N+ membrane (GE Healthcare bioscience, NJ, USA) according to manufacture's protocol. For Northern blot analysis of *rbcS*-siRNAs, small RNAs were separated on a 15% polyacrilamide-Tris-bolate-EDTA-urea gel and transferred to a Hybond-N+ membrane by electroblotting. After baking and UV-closslinking, the membranes were hybridized with digoxigenin (DIG)-labeled antisense RNA probes. For detection of ALSV-RNAs, Dig-labeled RNA probes complementary to positions 1 to 476 of ALSV-RNA1 (Genbank/EMBL/DDBJ accession no. AB030940) and 1 to 433 of ALSV- RNA2 (Genbank/EMBL/DDBJ accession no. AB030941) were used. DIG-labeled RNA probes complementary to positions 251 to 481 of *rbsS *was used for detection of *rbcS*-mRNA and *rbcS*-siRNAs. Prehybridization (2 h) and hybridization (18 h) were carried out at 68 °C (*rbcS*-mRNA) and 40 °C (*rbcS*-siRNAs) in a hybridization solution containing 50% formamide, 5xSSC, 2% blocking reagent (Roche Diagnostics, Basel, Switzerland), 0.1% sarcosyl, and 0.02% SDS. The membrane was washed twice for 5 min with 2xSSC, 0.1%SDS, and twice for 15 min with 0.1xSSC, 0.1% SDS at 68°C (*rbcS*-mRNA) and 40 °C (*rbcS*-siRNAs). Chemiluminescent detection was conducted by anti-digoxigenin-AP, Fab fragments (Roche Diagnostics) and CDP-Star Chemiluminescent substrate according the manufacturer's protocol. The membrane was then exposed to X-ray films.

For RT-PCR-Southern blot hybridization, total RNAs were isolated from shoot apices of three MdTFL-ALSV-infected apple seedlings about 40 days post inoculation(dpi). A specific primer for *MdTFL1 *(5'-GTGGCATACATTGTAAATA-3') [51] was used in a reverse transcription reaction with 500 ng of total RNA as a template. PCR reactions were run for 30 cycles at 50°C using a sense primer 2S (5'-CTCTTAAAATGAAAAGAGCC-3') and an antisense primer 2A (5'-CTCTAAAGAAGCCTTTAT-3') [51] in 50 μl PCR solution. PCR products (20 μl and 1 μl) were separately electrophoresed on two 1.5% agarose gels. A 20 μl-gel was staind with ethidium bromide and a 1 μl-gel was blotted on the Hybond-N+. Southern hybridization using DIG-labeled RNA probes specific for *MdTFL1 *sequence was carried out at 50°C. Washing and signal detection was performed as described above.

### In situ *hybridization*

Shoot apices of three ALSV-infected apple seedlings (No. 1, 2 and 3) after flowering were sampled and fixed in FAA (50% ethanol, 10% formalin, 5% acetic acid) for 5 h. After fixation, they were dehydrated through an incremental ethanol and lemozol series and embedded in Paraplast Plus (Sigma-Aldrich, StLouis, USA). Tissue sections were cut using a rotary microtome (Yamatokouki, Asaka, Japan) set to 10 μm, mounted on glass slides coated with APS (Matsunami, Osaka, Japan), and then baked on the slides at 48 °C for 24 h. The sections were deparaffinized by lemozol and rehydrated using a decreasing-concentration ethanol series. The sections were treated with proteinase K (1 μg ml^-1^), 4% paraformaldehyde, and then acetylated. DIG-labeled antisense RNA probe complementary to positions 241 to 441 of *MdTFL1*-mRNA were used for detection of *MdTFL1*-mRNA. DIG-labelled sense RNA probe was used as a control. Hybridization, colorigenic detection, and observation were performed as described by Nakamura *et al*. [46].

## List of Abbreviations

ALS: *Apple latent spherical virus; CPN60α *alpha subunit of chloroplast chaperonin; dpi: *Days post inoculation; EF-1a*: elongation factor 1 alpha; mpi: *months post inoculation*; *FT: FLOWERING LOCUS T; TFL1: TERMINAL FLOWER 1*; *rbcS*: riburose-1, 5-bisphosphate carboxylase small subunit; TRV: *Tobacco rattle virus*; wpi: *weeks post inoculation*.

## Competing interests

The authors declare that they have no competing interests.

## Authors' contributions

SS constructed the ALSV vectors used in this study. SS and NYA carried out the inoculation of the ALSV vectors to apple, pear, and Japanese pear. SS carried out the RT-PCR and Northern blot analysis. NYA carried out the *in situ *hybridisation analysis and RT-PCR-Southern blot analysis. NYO is the principal investigator, supervised the experiments and the writing of the manuscript. All authors read and approved the final manuscript.
